# Immediate versus delayed intramedullary nailing for open fractures of the tibial shaft: A multivariate analysis of factors affecting deep infection and fracture healing

**DOI:** 10.4103/0019-5413.43385

**Published:** 2008

**Authors:** Kazuhiko Yokoyama, Moritoshi Itoman, Masataka Uchino, Kensuke Fukushima, Hiroshi Nitta, Yoshiaki Kojima

**Affiliations:** Department of Orthopaedic Surgery, Machida Municipal Hospital, Machida, Tokyo, Japan; 1 School of Medicine, Kitasato University, Sagamihara, Kanagawa, Japan

**Keywords:** Deep infection, fracture healing, intramedullary nailing, multivariate analysis, open tibial fracture, predictive factors

## Abstract

**Background:**

The purpose of this study was to evaluate contributing factors affecting deep infection and fracture healing of open tibia fractures treated with locked intramedullary nailing (IMN) by multivariate analysis.

**Materials and Methods:**

We examined 99 open tibial fractures (98 patients) treated with immediate or delayed locked IMN in static fashion from 1991 to 2002. Multivariate analyses following univariate analyses were derived to determine predictors of deep infection, nonunion, and healing time to union. The following predictive variables of deep infection were selected for analysis: age, sex, Gustilo type, fracture grade by AO type, fracture location, timing or method of IMN, reamed or unreamed nailing, debridement time (≤6 h or >6 h), method of soft-tissue management, skin closure time (≤1 week or >1 week), existence of polytrauma (ISS< 18 or ISS≥18), existence of floating knee injury, and existence of superficial/pin site infection. The predictive variables of nonunion selected for analysis was the same as those for deep infection, with the addition of deep infection for exchange of pin site infection. The predictive variables of union time selected for analysis was the same as those for nonunion, excluding of location, debridement time, and existence of floating knee and superficial infection.

**Results:**

Six (6.1%; type II Gustilo n=1, type IIIB Gustilo n=5) of the 99 open tibial fractures developed deep infections. Multivariate analysis revealed that timing or method of IMN, debridement time, method of soft-tissue management, and existence of superficial or pin site infection significantly correlated with the occurrence of deep infection (*P*< 0.0001). In the immediate nailing group alone, the deep infection rate in type IIIB + IIIC was significantly higher than those in type I + II and IIIA (*P* = 0.016). Nonunion occurred in 17 fractures (20.3%, 17/84). Multivariate analysis revealed that Gustilo type, skin closure time, and existence of deep infection significantly correlated with occurrence of nonunion (*P* < 0.05). Gustilo type and existence of deep infection were significantly correlated with healing time to union on multivariate analysis (r^2^ = 0.263, *P* = 0.0001).

**Conclusion:**

Multivariate analyses for open tibial fractures treated with IMN showed that IMN after EF (especially in existence of pin site infection) was at high risk of deep infection, and that debridement within 6 h and appropriate soft-tissue managements were also important factor in preventing deep infections. These analyses postulated that both the Gustilo type and the existence of deep infection is related with fracture healing in open fractures treated with IMN. In addition, immediate IMN for type IIIB and IIIC is potentially risky, and canal reaming did not increase the risk of complication for open tibial fractures treated with IMN.

## INTRODUCTION

The treatment of open fractures of the tibial shaft remains controversial. The precarious blood supply and the lack of soft-tissue cover of the shaft of the tibia make these fractures vulnerable to nonunion and infection. Basic concepts of the current strategy for open tibial fractures reducing these complications are as follows: (1) immediate intravenous antibiotics; (2) urgent and repeated surgical debridement; (3) immediate rigid skeletal stabilization; (4) early, appropriate soft-tissue coverage, and (5) subsequent early bone grafting beneath a stable soft-tissue cover.^1^–^3^

Moreover, there have been still various questions or problems in the treatment as follows: What kind of stabilization, such as external fixation (EF), intramedullary nailing (IMN), plating, or Ender nailing, is appropriate for immediate stabilization in open tibial fractures?^4^–^8^ Whether reamed or unreamed nailing is appropriate in the use of IMN?^7^,^9^ What kind of soft-tissue injury grade is the limit in immediate IMN?^5^,^7^,^9^,^10^ When should soft-tissue cover be performed?^2^,^11^ When is appropriate conversion to IMN from EF?^12^,^13^

In the point of view of evidence-based medicine (EBM) for the resolution of the above problems, retrospective case–control cohort study, randomized controlled trial (RCT) study, multicenter study, and meta-analysis are more effective. However, it is difficult to perform the above trials because of its cost or ethics problem in randomization. Multivariate analysis in retrospective study could reduce several biases in clinical setting. The objective of the present study is to evaluate factors affecting deep infections and fracture healing of an open fracture of the tibial shaft treated with immediate or delayed locked IMN, using multivariate analysis in retrospective setting.

## MATERIALS AND METHODS

### Clinical materials

Our institutional review board approved this study. This study was a retrospective chart and radiographic review. From existing records, we identified 98 consecutive cases with 99 open tibial shaft fractures that underwent immediate or delayed locked IMN, between 1991 and 2002. These operations were performed under the direction of six orthopedic staff doctors and four staff doctors in the Department of Plastic and Reconstructive Surgery.

Eighty-four patients were male, and 14 patients were female. The mean age of the patients at the time of injury was 34.6 years (range, 15–86 years). Patients were divided into three groups on the basis of age as follows: 70 fractures belonged to group 1 aged 45 years or younger, 20 fractures to group 2 aged 46–59 years, and nine fractures to group 3 aged 60 or older.

Ninety patients were injured in motor vehicle accidents (53 were injured in motorcycle accidents, 21 were passengers or drivers in cars, 12 were pedestrians struck by automobiles, and four were injured bicycle drivers struck by automobiles), four patients were injured in labor accidents, and the remaining four patients were injured in other causes.

The mean Injury Severity Score (ISS)^14^ of the 98 patients was 12.8 (range, 9–27). Patients were divided into two categories according to the criteria of Bone *et al*.,^15^ to distinguish between multiple injured patients and those with multiple skeletal injuries alone (additional fracture of the femur, tibia, humerus, forearm, simple pelvis or spine, without cord injury): 74 fractures had an ISS ≥ 18, and 25 had an ISS ≥ 18. All patients with multiple skeletal injuries alone had an ISS < 18.

The 99 open tibial fractures were classified according to the criteria of Gustilo *et al*.^16^,^17^: type I, 22 fractures; type II, 42 fractures; type IIIA, 13 fractures; type IIIB, 20 fractures, and type IIIC, two fractures. We used Hannover Fracture Scale '98 (HFS)^18^ to distinguish another grading of soft-tissue injury, although HFS was devised for predictive indices of limb salvage or amputation using a clear point system. The mean HFS scores in types I, II, IIIA, IIIB, and IIIC were 1.1, 1.5, 2.9, 6.9, and 9.0, respectively. The order of the mean HFS in respective Gustilo type was as follows: type 1 = type II < type IIIA < type IIIB = type IIIC (*P* < 0.01 in one-way analysis of variance (ANOVA) and post hoc Tukey's test). Accordingly, the open tibia fractures were divided into thee groups as following: type I + II, IIIA, and IIIB + IIIC.

Severity of fractures was classified according to AO/ASIF classification^19^: type A, 49 fractures; type B, 39; type C, 11. One fracture was in the proximal third of the tibial shaft; 91 were in the middle third; four were in the distal third, and three were segmental fractures.

### Treatment and group

**Figure 1:**
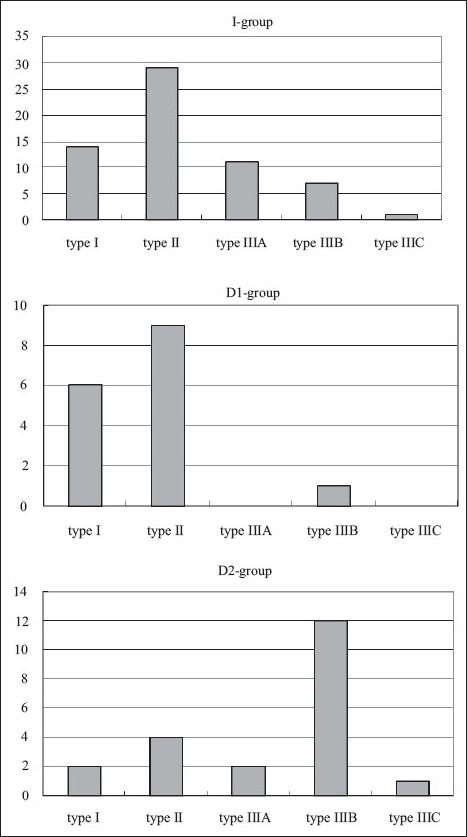
Bar diagram showing the distribution of Gustilo type in each intramedullary nailing treatment group

Patients were divided into the following three treatment groups according to the timing and circumstances of IMN: group I, immediate IMN at the time of initial debridement (n = 62); group D1, delayed IMN following nonoperative treatment such as skeletal traction or splint (n = 16); group D2, delayed IMN following external fixation (n = 21). Group D2 was subdivided into group D2a: planned conversion (n = 17); and group D2b: nailing for established nonunion treated with EF (n = 4). Conversion from external fixation to IMN was performed, if no bacteria are found in smears from the open wound and the entire pin site area immediately before the second operation. The mean stabilization times in groups D1 and D2 were 18.3 ± 3.0 days (mean ± SE) and 52.1 ± 16.0 days, respectively. Unreamed procedures in these IMNs were performed for 63 fractures (63.6%). The distribution of Gustilo type in each treatment group was shown in Figure 1. There were many cases of type II in group I. There were many cases of type I and II in group D1. And there were many cases of type IIIB in group D2. In addition, the mean HFS scores in groups I, D1, and D2 were 2.2 ± 0.27, 1.9 ± 0.44, and 5.3 ± 0.73, respectively. Although there was no significant difference between the mean HFS scores in group I and that in group D1, the mean HFS score in group D2 was significantly higher than those in groups D1 and I (*P* < 0.001 in one-way ANOVA and post hoc Tukey's test).

No protocol was followed for deciding between immediate and delayed IMN, or for deciding between reamed and unreamed IMN. Decisions between immediate and delayed IMN and between reamed and unreamed IMN were based on the experience and discretion of the attending orthopedic staff, the interval since injury, the degree of contamination of the wound, the extent of injury to the soft tissues, and the degree of associated vital organ injuries. Kitasato Cylinder Nails (Mizuho Inc., Tokyo, Japan) manufactured at our institution in 1979^20^ and AO/ASIF unreamed Tibial Nails (Synthes Inc./Mathys Medical Ltd., Japan, Tokyo, Japan) were used for reamed and unreamed IMN, respectively. Reamed IMN was performed with as limited reaming technique.^21^ All fractures were initially stabilized by static locking.

Intravenous antibiotic treatment with a first degree cephalosporin for Gustilo type I and II fractures with the addition of an aminoglycoside (usually gentamicin) for type-III fractures was begun in the emergency room, and continued for 72–96 hours after the initial procedure. After the patient was resuscitated and all required emergency surgical procedures were completed, the open wound was irrigated and debrided. Irrigations were performed by using low-pressure bulb syringes to type I and II fractures, and performed by using high-pressure pulsating water jet devices to type III fractures. Debridement was repeated at 48-hour intervals until the wound was clean and all devitalized tissue had been resected.

**Figure 2:**
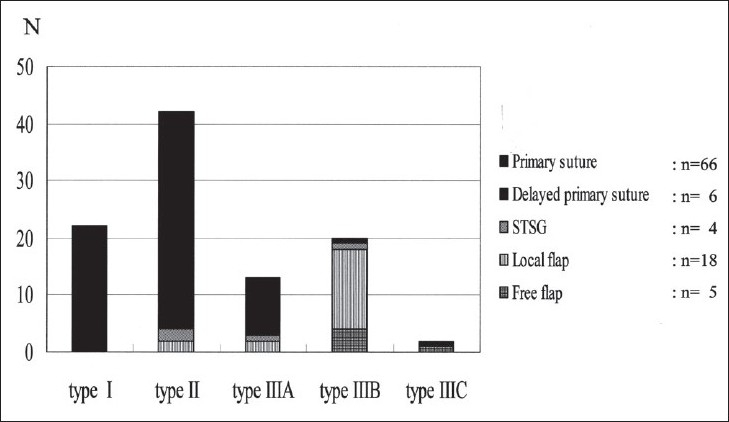
Bar diagram showing the distributions of soft-tissue management in each Gustilo are shown. The primary sutures were performed in many cases of type I and II. The soft-tissue management in type IIIA consisted of primary sutures or delayed primary sutures. That in type IIIB consisted of local or free flaps. STSG: Split-thickness skin grafting.

**Figure 3:**
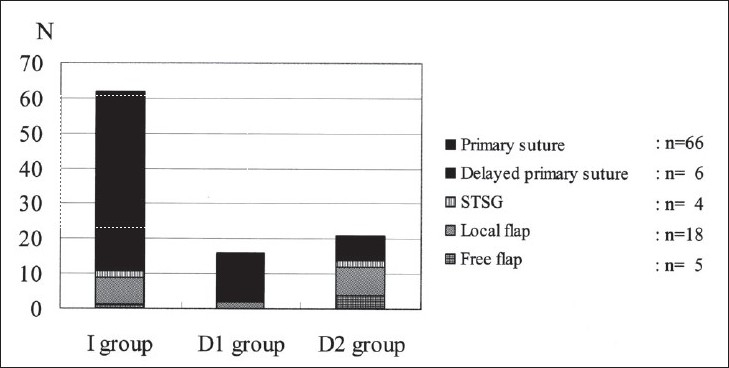
Bar diagram showing the distributions of soft-tissue management in each intramedullary nailing group are shown. The soft-tissue management in treatment group I and D1 consisted of primary sutures or local flaps. And the soft-tissue managements in group D2 consisted of primary sutures and local or free flaps. STSG: Split-thickness skin grafting.

The soft-tissue managements in the present series were as follows: primary suture (n = 66; 66.7%); delayed primary suture (n = 6; 6.1%); secondary split-thickness skin grafting (STSG) (n = 4; 4.0%); local flap (n = 18; 18.2%); and free flap (n = 5; 5.1%). The distributions of soft-tissue management in each Gustilo type and each IMN group are shown in Figures 2 and 3. The primary sutures were performed in many cases of types I and II. The soft-tissue management in type IIIA consisted of primary sutures or delayed primary sutures, and that in type IIIB consisted of local or free flaps. The soft-tissue management in treatment group I and D1 consisted of primary sutures or local flaps and in group D2 consisted of primary sutures and local or free flaps.

### Evaluations and statistical analysis

Follow-up evaluations lasted from 1.6 to 10 years (mean, 3.1 years) after the original injury. Medical records of all patients were reviewed in detail. Roentgenograms were available for 84 fractures. The union time could be calculated in 78 fractures (79%). Union time in the remaining six cases could not be clearly obtained, because the status of bony union had already been consolidated at the visit to our outpatient clinic including taking plain radiographic films. Several senior staff doctors, who were double-blinded with respect to the detail of respective cases, performed the evaluations of the fracture healing time.

The deep, superficial, and pin site infection rates (in D2 group alone), nonunion rate, and time to union were assessed on the basis of clinical charts and radiographs. In addition, each case belonging to group D2b was excluded in the fracture healing analysis. Superficial and deep infections were defined according to Dellinger *et al*.^22^ A superficial wound infection was located entirely above fascia with erythema and tenderness that required antibiotic therapy and opening the wound. A deep infection involving bone was defined as infection involving tissue below the muscular fascia. Pin tract infection was defined as any persistent drainage from a pin site requiring intervention or positive bacterial cultures from the pin site.^1^,^10^ Bony union was defined as follows: clinically, there was no pain or tenderness, and the patient walked without aids; radiographically, solid bridging callus had connected the fracture fragment on both anteroposterior view and lateral view.^23^ Nonunion was defined as a lack of clinical or radiographic evidence of healing 12 months after the injury, requiring a secondary procedure.

The three measures of final outcome were deep infection, nonunion, and healing time to union. Categorical regression analyses were performed if *P* values in chi-square test or Fisher's exact test showed less than 0.05 to determine predictors of the former two outcomes. Multiple regression analysis (stepwise method) after unpaired t-test or one-way ANOVA combined with Tukey's post hoc test was derived to determine predictors of the healing time to union.

The following predictive variables of deep infection were selected for analysis, based on our speculation that they contribute to deep infection: age (≤45 years, 46–49 years, ≥60 years), sex (male or female), Gustilo type (I + II, IIIA, or IIIB + IIIC), fracture location (proximal, middle, distal, proximal + middle, or middle + distal), fracture grade (AO type: A, B, or C), debridement time (≤6 h or >6 h), treatment type 1 (I group, D1 group, D2a group, or D2b group), reaming (reamed or unreamed), method of soft-tissue management, skin closure time (≤1w or >1w), ISS (< 18 or ≥18), superficial infection (+ or −), pin site infection (+ or −), and floating knee injury (+ or −).

For the analysis of predictive variables of nonunion, we selected the factors used for analysis of deep infection, minus pin site infection, plus the following factors: treatment type 2 (I group, D1 group, or D2a group) and existence of deep infection (+ or −). We excluded D2b group because of cases in need of nailing due to the existence of clear nonunions.

For the analysis of predictive healing time to union, we selected age, sex, Gustilo type, fracture grade (AO type), treatment type (I group, D1 group, or D2 group), existence of reaming, method of soft-tissue management, skin closure time, ISS, and existence of deep infection, similar to predictive analysis of nonunion.

The above categorical and numerical data were used for several univariate analyses, categorical regression analysis, or multiple regression analysis, which was performed using SPSS 11.0 for Windows (SPSS, Chicago, IL, USA) and a personal computer. The regression coefficient for individual predictors of the three outcomes were calculated, and a *P* value of <0.05 was considered to indicate significance.

## RESULTS

### Deep infections

Six (6.1%) of the 99 open tibial fractures developed deep infections. Three of these infections were caused by methicillin-resistant Staphylococcus aureus (MRSA); two were caused by Pseudomonas aeruginosa, and one was caused by both. The deep infections occurred in one Gustilo type II (1.0%, 1/99) and five type IIIB (5.1%, 5/99).

The relationship between deep infection and the following factors was evaluated: Gustilo type, treatment type 1, debridement time, method of soft-tissue management, skin closure time, existence of superficial infection, and existence of pin tract infection showed *P* < 0.05 on univariate analysis, among examined factors. Then, the relationship between the occurrence of deep infection and the above significant predictive factors was evaluated by means of categorical regression analysis. Finally, the above five factors excluding skin closure time were significant factors affecting the occurrence of deep infection on multivariate analysis (*P* = 0.0001). The orders of significant factors were as follows: (1) treatment type 1 and method of soft-tissue management, (2) existence of superficial infection, (3) debridement time, and (4) existence of pin tract infection. The statistical results about deep infection analysis are summarized in Table 1. In addition, the deep infection rates of type I + II, type IIIA, and type IIIB + IIIC in immediate IMN group alone were 2.3% (1/43), 0% (0/11), and 25% (2/8), respectively. The deep infection rate in type IIIB + IIIC was significantly higher than those in type I + II and IIIA (*P* = 0.016).

**Table 1:** Deep infection rate, *P* value in univariate analysis, and regression coefficient and *P* value in categorical regression analysis in each factor

Factor		Deep infection rate (%)	*P* value in univariae analysis	Regression coefficient	*P* value in categorical regression analysis
Age	≤45 yrs	5.7 (4/70)	0.57	0.195	0.068
	46-59 yrs	10 (2/20)			
	≥60 yrs	0 (0/9)			
Sex	Male	7 (6/85)	0.31		
	Female	0 (0/14)			
Gustilo type	I + II	1.6(1/64)	0.001*	0.195	0.068
	IIIA	0 (0/13)			
	IIIB + IIIC	22.7 (5/22)			
AO type	A	2 (1/49)	0.11		
	B	7.7 (3/39)			
	C	18.2(2/11)			
Site	Proximal site (P)	0 (0/1)	0.59		
	Middle site (M)	5.5 (5/91)			
	Distal site (D)	25 (1/4)			
	P+M	0 (0/2)			
	M + D	0 (0/1)			
Treatment type 1	I group	4.8 (3/62)	0.002*	0.458	0.0001*
	D1 group	0 (0/16)			
	D2a group	5.9 (1/17)			
	D2b group	50 (2/4)			
R vs UR	R	8.3 (3/36)	0.67		
	UR	4.8 (3/63)			
Debridement time	≤6	4.5 (4/89)	0.049*	0.202	0.005*
	>6	25 (2/10)			
Method of soft-tissue management	Primary suture	1.5 (1/66)	0.001*	0.278	0.0001*
	Delayed primary suture	16 (1/6)			
	STSG	0 (0/4)			
	Local flap	11.1 (2/18)			
	Free flap	40 (2/5)			
Skin closure time	≤1w	3.3 (3/90)	0.01*	0.89	0.423
	>1w	33.3 (3/9)			
Multiple trauma	ISS<18	8.1 (6/74)	0.33		
	ISS≥18	0 (0/25)			
Floating knee	-	6 (5/84)	1.0		
	+	6.7 (1/15)			
Sup inf	-	4.3 (4/94)	0.001*	0.22	0.003*
	+	40 (2/5)			
Pin site inf	-	13.6 (3/22)	0.001*	0.415	0.014*
	+	100(1/1)			
	NR	2.6 (2/76)			

Inf-infection, ISS-Injury Severity Score, NR-Non-related, R-Reamed, sup-superficial, STSG-Split Thickness Skin Graft, UR-Unreamed

* -*P* value<0.05. Underlined *P* values in univariate analysis show those evaluated by Fisher's exact test

From the above result, it was postulated that, for open tibial fractures, IMN after EF (especially in existence of pin site infection) was at high risk of deep infection; debridement within 6 h and appropriate soft-tissue managements were also important factors in preventing deep infections. Immediate IMN for type IIIB and IIIC is potentially risky, and canal reaming did not increase the risk of complication for open tibial fractures, irrespective of immediate or delayed IMN.

### Fracture healing

Seventeen (20.3%) of the 84 open tibial fractures developed nonunions The relationship between nonunion rate and the following factors: Gustilo type, method of soft-tissue management, skin closure time, existence of superficial infection, and existence of deep infection showed *P* < 0.05 on univariate analyses, among examined factors. Then, the relationship between the occurrence of nonunion and the above significant predictive factors were evaluated by means of categorical regression analysis. Finally, the above three factors, such as Gustilo type, skin closure time, and existence of deep infection, were significant factors affecting the occurrence of nonunion on multivariate analysis (*P* < 0.05). The orders of significant factors were as follows: (1) Gustilo type and existence of deep infections and (2) skin closure time. The statistical results about nonunion analysis are summarized in Table 2.

**Table 2:** Nonunion rate, *P* value in univariate analysis, and regression coefficient and *P* value in categorcial regression analysis in each factor

Factor	Nonunion rage (%)	*P* value in univariate analysis	Regression coefficient	*P* value in categorical regression analysis
Age	≤45 yrs	17.5 (11/63)	0.28		
	46-59 yrs	35.7 (5/14)			
	≥60 yrs	14.3 (1/7)			
Sex	Male	20 (14/70)	1.0		
	Female	21.4 (3/14)			
Gustilo type	I + II	9.4 (5/53)	0.001*	0.473	0.0001*
	IIIA	9.1 (1/11)			
	IIIB +IIIC	55 (11/20)			
AO type	A	12.2 (5/41)	0.077	0.062	0.672.
	B	23.5 (8/34)			
	C	44.4 (4/9)			
Site	Proximal site (P)	0 (0/1)	0.65		
	Middle site (M)	21.8 (17/78)			
	Distal site (D)	0 (1/4)			
	P+M	0 (0/3)			
	M+D	0 (0/2)			
Treatment type 2	I group	21.6 (11/51)	0.24		
	D1 group	6.3 (1/16)			
	D2a group	29.4 (5/17)			
R vs UR	R	22.2 (12/54)	0.67		
	UR	16.7 (5/30)			
Debridement time	≤6	18.9 (14/74)	0.42		
	>6	30 (3/10)			
Method of soft-tissue management	Primary suture	9.3 (5/54)	0.008*	0.1891	0.05
	Delayed primary suture	33.3 (2/6)			
	STSG	0 (0/2)			
	Local flap	44.4 (8/18)			
	Free flap	50 (2/4)			
Skin closure time	≤1w	16.9 (13/77)	0.03*	0.194	0.026*
	>1w	57.1 (4/7)			
Multiple trauma	ISS<18	21.9 (14/64)	0.33		
	ISS≥18	15 (3/20)			
Floating knee	-	17.1 (12/70)	1.0		
	+	35.7 (5/14)			
Sup inf	-	17.7 (14/79)	0.024*	0.005	0.99
	+	60 (3/5)			
Deep inf	-	16.3(13/80)	0.001*	0.329	0.0001*
	+	100			

inf-infection, ISS-Injury Severity Score, R-Reamed, sup-superficial, STSG-Split Thickness Skin Graft, UR-Unreamed

* *P* value<0.05. Underlined *P* values in univariate analysis show those evaluated by Fisher's exact test

The relationship between fracture healing time and the following factors: Gustilo type, skin closure time, and existence of deep infection showed *P* < 0.05 on univariate analyses, among examined factors. Moreover, the relationship between the healing time and all the above evaluated factors was evaluated by means of multiple regression analysis (stepwise method). Finally, the two factors, namely Gustilo type and existence of deep infection, were significantly correlated with healing time to union on multivariate analysis (r^2^ = 0.263, *P* = 0.0001). The predictive regression equation for time to union was as follows: healing time to union = –4.20 + 11.14 existence of deep infection + 2.28 × Gustilo type. The statistical results about union time analysis are summarized in Table 3.

**Table 3:** Union time, *P* value in univariate analysis, and regression coefficient and *P* value in multiple regression analysis in each factor

Factor		Union time (months)	*P* value in univariate analysis	Regression coefficient	*P* value in multiple regression analysis
Age	≤45 yrs	10.6 ± 0.9 (n=59)	0.26§	
	46-59 yrs	14.3 ± 2.5 (n=13)			
	≥60 yrs	12.2 ± 1.82 (n=6)			
Sex	Male	11.7 ± 1.0 (n=65)	1.0¶		
	Female	9.8 ± 1.4 (n=13)			
Gustilo type	I + II	9.6 ± 0.7 (n=49)	0.001*§	2.28	0.0001*
	IIIA	8.6 ± 1.2 (n=9)			
	IIIB+IIIC	16.7 ± 2.4 (n=20)			
AO type	A	10.1 ± 0.8 (n=38)	0.13§		
	B	12.0 ± 1.7 (n=30)			
	C	14.3 ± 2.5 (n=10)			
Treatment type 3	I group	10.7 ± 0.9 (n=47)	0.12§		
	D1 group	9.4 ± 1.5 (n=13)			
	D2a group	14.4 ± 2.6 (n=18)			
R vs. UR	R	11.6 ± 1.1 (n=50)	0.85¶		
	UR	11.2 ± 1.4 (n=28)			
Method of soft-tissue management	Primary suture	10.2 ± 1.0 (n=49)	0.064§		
	Delayed primary suture	11.6 ± 3.8 (n=5)			
	STSG	6.2 ± 1.0 (n=4)			
	Local flap	13.1 ± 1.6 (n=17)			
	Free flap	21.3 ± 7.8 (n=3)			
Skin closure time	≤1w	10.7 ± 0.8 (n=70)	0.015*¶		
	>1w	17.4 ± 3.3 (n=8)			
Multiple trauma	ISS<18	11.4 ± 1.0 (n=61)	0.95¶		
	ISS≥18	11.2 ± 1.6 (n=17)			
Deep inf.	-	10.5 ± 0.8 (n=73)	0.041*¶	11.1	0.0001*
	+	24.0 ± 4.6 (n=5)			

inf-infection, ISS-Injury Severity Score, R- Reamed, STSG- Split Thickness Skin Graft, UR- Unreamed, Union time-expressed as mean ± SEM

§ *P* value evaluated by one-way ANOVA and post hoc test

¶ *P* value evaluated by unpaired *t*-test

* *P* value<0.05

From both the nonunion analysis and union time assay, it was summarized that both Gustilo type and existence of deep infection were associated with fracture healing.

## DISCUSSION

Currently, immediate and repeated wound debridement, urgent and rigid skeletal stabilization, and early wound coverage combined with antibiotic therapy are the preferred treatment for open tibial fractures. However, the methods used for skeletal stabilization of these injuries remains controversial, with several options such as bone plates, intramedullary rods, external fixations, and IMN.^4^–^10^

Among the above stabilizations, there have been several prospective comparison studies between interlocking IMNs and EFs for open tibial fractures.^5^,^7^ Henley *et al*.^5^ compared unreamed IMN with EF in patients with type II, IIIA, and IIIB open fractures of the tibial shaft. They concluded that unreamed interlocking intramedullary nails were more efficacious than half-pin external fixators, with regard to maintenance of limb alignment. They also mentioned that the severity of soft-tissue injury rather than the choice of implant appeared to be the predominant factor influencing rapidity of bone healing and rate of injury site infection. However, the present study revealed that the severity of soft-tissue injury (Gustilo type) was not significantly related to the incidence of deep infections in open tibial fractures treated with IMN.

Moreover, the debate has focused on whether IMN should be performed with or without reaming. Several authors^9^,^10^,^24^ have failed to clearly identify any differences with regard to rates of infection or nonunion or functional outcome between reamed IMN regimen and unreamed IMN regimen. Bhandari *et al*.^25^ also failed to demonstrate any significant differences with regard to infection, nonunion, or reoperations. Currently, it is not possible to make a recommendation for or against reaming in the fixation of open tibial fractures, according to a most recent review.^26^ In our study also, canal reaming did not increase the risk of complication for open tibial fractures treated with IMN.

The present multivariate analyses about infections in open tibial fractures treated with immediate and delayed IMN showed that IMN after EF (especially in existence of pin site infection) was at high risk of deep infection as a first conclusion, and that debridement within 6 h and appropriate soft-tissue management were also important factors in preventing deep infections as a second conclusion. As a third conclusion, it was shown that immediate IMN for type IIIB and IIIC was potentially risky.

In managing severe open tibial fractures initially treated with external fixations, several surgeons, including us,^13^,^26^ performed conversion IMN from external fixators to decrease various complications, such as delayed unions, nonunions, malunions, and ankle joint stiffness. However, some authors^27^,^28^ reported a high incidence of deep infection in secondary or delayed IMN after external fixation. Bhandari *et al*.^29^ concluded in a meta-analysis study about this conversion method for tibial fractures that the existence of pin site infections was the most important factor in the expansion of infections, which is the same result as in our study.

Many trauma surgeons have mentioned comments similar to our second conclusion, i.e., debridement within 6 h and appropriate soft-tissue managements as important factors protecting from deep infections in open fractures. Generally speaking, urgent debridement for open fractures should be performed within 6 h after original injury. However, the origin of so-called 6-h rule is unclear. A number of studies have called the 6-h rule into question.^30^–^32^ A few authors^33^ have gone so far as to suggest that operative debridement might not be necessary for low-grade open fractures. Our basic concept, however, is that operative debridement should be considered as the standard care for all open fractures. Even if the benefits of standard debridement were found to be insignificant for low-grade open fractures, operative debridement is required for appropriate classification of wounds.

Although skin closure time (less than or more than 7 days) is significant in the present univariate analysis, this factor is not significant in this multivariate analysis. Several authors^2^,^11^,^34^–^36^ have documented significantly better outcomes with early closure (within 7 days). The timing of soft-tissue coverage in open fractures remains controversial.^3^,^28^,^34^–^38^ Osterman *et al*.^38^ reported that wound closure should be obtained within 7 days to prevent infectious complications in severe open fractures managed with the antibiotic bead pouch technique and stabilization by external fixators. Recently, a radical immediate or very early fix and flap (within 24–72 h) protocol has been developed, based on a close collaboration between the orthopedic and microsurgical teams by Gopal *et al*.^39^ Their report on such management showed a rate of union of 100% although with delayed union in 62% and a rate of infection of 9.5%. On the other hand, Hertel *et al*.^37^ concluded that immediate soft-tissue reconstruction in association with immediate radical debridement and early definitive skeletal stabilization was the timing of choice for soft-tissue coverage in severe lower leg fractures if the general condition of the patient permitting. Anyway, the issue of infections related to soft-tissue coverage timing can depend on various factors, such as, coverage method, stabilization method, soft-tissue injury itself, and other organ injuries. In other words, it is difficult to conclude these problems briefly and simply.

The borderline of soft-tissue severity in reamed or unreamed IMN for open tibial fractures has not yet been solved clearly. Sanders *et al*.^40^ indicated that unreamed tibial nailing was an acceptable technique for use in all open tibial shaft fractures (excluding type IIIC). They also reported that the overall chronic infection rate was 4%, with no infections in type I, II, and IIIA open fractures and a 13% rate in type IIIB open fractures. Moreover, Gaebler *et al*. reported in a multicenter analysis of closed and open tibial fractures treated with unreamed nailings that grade III fractures were serious injuries with an odds ratio 22.4 times higher of having a deep infection compared with patients with closed, grade I and II open fractures.^41^ However, no detailed information about subgroups of grade III was described. In a more recent prospective analysis of immediate unreamed IMNs for open tibial fractures (n = 143), the deep infection rate for the patients who were treated by protocol was only 3% (grade I: 1/33; grade II: 2/57, grade IIIB: 1/15).^42^ However, there were a small number of grade IIIB open tibial fractures in their series, and the validity of immediate IMN for severe open tibial fractures would not be clarified. Immediate IMN for IIIB and IIIC tibial fractures might be dangerous with respect to infection troubles on the basis of the above studies and the present analysis.

Our analyses postulated that both the Gustilo type and the existence of deep infection were associated with fracture healing in open fractures treated with IMN. Henley *et al*.^5^ described, as previously mentioned in the initial section of discussion, that the severity of the soft-tissue injury rather than the choice of implant appeared to be the predominant factor influencing the rapidity of bone healing. Their results were similar to our results. In most of the reports about fracture healing in IMN for open tibial fractures, the use of unreamed, small-diameter nail resulted in delayed union or implant failure associated with delayed fracture healing, when compared with the use of reamed nailing.^9^,^10^,^25^ However, reamed or unreamed procedures for open tibial fractures did not affect fracture healing in our multivariate analyses.

Lastly, we must mention an important issue in discussing the problem of treatment for open fractures. The Gustilo and Anderson classification is based on subjective description and is not an objective criterion based on scoring system. Two authors^43^,^44^ have found this classification system to be associated with low interobserver agreement. According to Brumback *et al*.,^43^ interobserver agreement in the classification of open fractures of the tibia was about 60% based on 9 results of a survey of 245 orthopedic surgeons. Thus, the reliability of this classification system has been problematic. A recent classification based on an objective scoring system composed of the Ganga Hospital Injury Severity Score is thought to be useful for predicting the occurrence of deep infection and functional outcome.^45^

Although HFS^18^ has been devised for predictive indices of limb salvage or amputation, this scale is composed of eight objective items ([1] bone loss, [2] skin injury, [3] muscle injury, [4] wound contamination, [5] deperiostation, [6] local circulation, [7] systemic circulation, and [8] neurology), using a clear point system. We previously reported a new scoring system based on this HFS for predicting the occurrence of deep infection in open tibial fractures.^46^ Thus, we reclassified our cases according to HFS, as another classification tool. In addition, we should evaluate the validity and reliability of a treatment and regimen for open fractures under the consideration of the above.

In conclusion, multivariate analyses for open tibial fractures treated with IMN showed that IMN after EF (especially in existence of pin site infection) was at high risk of deep infection, and that debridement within 6 h and appropriate soft-tissue management were also important factors in preventing deep infections. These analyses postulated that both the Gustilo type and the existence of deep infection were associated with fracture healing in open fractures treated with IMN. Moreover, immediate IMN for type IIIB and IIIC is potentially risky, and canal reaming did not increase the risk of complication for open tibial fractures treated with IMN.
